# Structural and functional properties of NH_2_-terminal domain, core domain, and COOH-terminal extension of αA- and αB-crystallins

**Published:** 2011-08-31

**Authors:** C.O. Asomugha, R. Gupta, O.P. Srivastava

**Affiliations:** Department of Vision Sciences, University of Alabama at Birmingham, Birmingham, AL

## Abstract

**Purpose:**

The purpose of the present study was to determine the biophysical and chaperone properties of the NH_2_-terminal domain, core domain and COOH-terminal extension of human αA- and αB-crystallins and correlate these properties to those of wild type (WT) αA- and αB-crystallins.

**Methods:**

WT αA- and αB-crystallins cloned into pET 100D TOPO vector, were used as templates to generate different constructs encoding specific regions (NH_2_-terminal domain [NTD], core domain [CD], and COOH-terminal extension, [CTE]). The specific regions amplified by PCR using plasmid DNA from WT αA and WT αB were: αA NTD (residues 1–63), αA CD (residues 64–142), αA CTE (residues 143–173), αB NTD (residues 1–66), αB CD (residues 67–146), and αB CTE (residues 147–175). Resultant blunt-end PCR products were ligated to a pET 100 Directional TOPO vector. DNA sequencing results confirmed the desired constructs. Positive clones were transformed into the BL21 Star (DE3) expression cell line. Protein expression and solubility were confirmed by SDS–PAGE and western blot analysis using a monoclonal antibody against a 6× His-tag epitope. Proteins were purified using Ni^2+^-affinity column chromatography, under native or denaturing conditions, and used for biophysical and chaperone function analyses.

**Results:**

A total of five constructs were successfully generated: αA NTD, αA CD, αB NTD, αB CD, and αB CTE. SDS–PAGE and western blot analyses showed that αA CD and αB CD were present in both the soluble and insoluble fractions, whereas mutant preparations with NTD alone became insoluble and the mutant with CTE alone became soluble. All purified constructs showed alterations in biophysical properties and chaperone function compared to WT α-crystallins. αA NTD and αB CTE exhibited the most notable changes in secondary structural content. Also, αA NTD and all αB-crystallin constructs showed altered surface hydrophobicity compared to their respective WT α-crystallins.

**Conclusions:**

Although the individual α-crystallin regions (i.e., NH_2_-terminal domain, core domain, and COOH-terminal extension) exhibited varied biophysical properties, each region alone retained some level of chaperone function. The NH_2_-terminal domains of αA and αB each showed the maximum chaperone activity of the three regions with respect to their WT crystallins.

## Introduction

Crystallin proteins are the major components of the mammalian lens fiber cell and are subdivided into three classes, α, β, and γ. The crystallins help to increase the refractive power of the lens and maintain lens transparency by forming high concentrations of soluble oligomers and participating in short-range interactions among themselves [1,2]. Of the crystallins, α-crystallin accounts for almost half of the total lens protein and its two homologous subunits, αA- and αB-crystallin, oligomerize to form hetero-oligomers of ~800 kDa in a 3:1 ratio in vivo [2-4]. α-Crystallin is also a member of the small heat shock protein (sHSP) family, which are stress-induced proteins of 12–40 kDa subunits containing a conserved α-crystallin domain of ~90 amino acids and flanked on either side by a variable hydrophobic NH_2_-terminal domain and a short, hydrophilic unstructured COOH-terminal extension with a conserved sequence motif [3-8].

Like other sHSPs, α-crystallins posses chaperone-like function [9], whereby they recognize and bind destabilized and improperly folded proteins [7,8]. However, α-crystallins are ATP-independent chaperones, and cannot refold proteins [6], but sequester aberrant proteins to prevent subsequent formation of light-scattering aggregates. Because there is no protein turnover in mature fiber cells of the lens [10], crystallins must survive the lifetime of the lens and transparency must be maintained despite environmental insults and post-translational modifications (PTMs) that occur with age. In this manner, α-crystallins play a critical role in the maintenance of lens transparency.

It has been well established that chaperone activity of α-crystallins is dependent upon oligomerization of α-crystallins and substrates through hydrophobic contacts between accessible hydrophobic surfaces of α-crystallin and exposed hydrophobic surfaces of unfolding proteins [9,11]. However, the mechanism of chaperone binding is still not fully understood and studies have been undertaken to determine which sites of α-crystallin are involved in its chaperone function. Many have attributed chaperone binding to hydrophobic sequences in the α-crystallin domain, as well as in the NH_2_-terminal domain (reviewed in [3,6,7]). Specifically, studies suggest a functional chaperone substrate-binding site resides in residues 70–88 of αA-crystallin [12], as well as residues 73–92 of αB-crystallin [13]. It has also been suggested that residues 54–61 of αB-crystallin play an essential role in αB chaperone activity, though unessential for target protein binding [14]. However, others have identified several substrate-binding sites in αB-crystallin, including two NH_2_-terminal domain sequences, four α-crystallin domain sequences, and one COOH-terminal extension sequence, using protein pin arrays [15].

Mutagenesis, in general, has been used as a tool to identify regions of α-crystallin necessary for chaperone function. Studies have generated “point mutations” as well as “truncation” mutations mimicking naturally occurring PTMs to study their affects on crystallin structure and function [16,17]. It has been shown that hereditary point mutations in αA-crystallin (R116C) and αB-crystallin (R120G) cause structural and functional alterations that lead to congenital cataract and desmin-related myopathy, respectively [18,19]. Using site-directed mutagenesis, we mimicked commonly occurring age-related deamidations in αA- and αB-crystallin and demonstrated that N123D deamidation in αA-crystallin and N146D deamidation in αB-crystallin are crucial for chaperone activity [20,21]. We also performed a comprehensive study comparing two PTMs, deamidation and truncation, and showed that N123 deamidation in αA-crystallin [22] and N146 deamidation in αB-crystallin (Unpublished), as well as truncation of NH_2_- and COOH-termini, were detrimental to chaperone function. Whether mutations in the NH_2_-terminal domain, α-crystallin core domain, or the COOH-terminal extension of α-crystallin [23-27] affect both structure and function of the protein remains unclear. Previous studies have reported that removal/swapping of residues/regions from either the NH_2_-terminal domain or the COOH-terminal extension in both αA- and αB-crystallins cause improper folding and reduced chaperone activity [28-31], however none of these studies have explored the structural/functional stability of the three individual regions (i.e., NH_2_-terminal domain, core domain and COOH-terminal extension) of αA- and αB-crystallins.

With all the variations in results in the literature, it is still unclear whether synergistic effects of the three regions or their specific peptides are responsible for the chaperone activity of αA- and αB-crystallins. In an attempt to better understand this phenomenon of which of the three regions is most important for chaperone activity of αA- and αB-crystallins, we examined each region (NH_2_-terminal domain, core domain, and COOH-terminal extension) individually. The following constructs: αA NH_2_-terminal domain (αA NTD, residues 1–63), core domain (αA CD, residues 64–142) and αB NH_2_-terminal domain (αB NTD, residues 1–66), core domain (αB CD, residues 67–146), and COOH-terminal extension (αB CTE, residues 147–175) were generated and used to determine the structure and function of the individual regions and narrow down which region is most pertinent to chaperone function. Our results reveal that despite structural alterations in proteins representing the NH_2_-terminal domain, core domain, and COOH-terminal extension, compared to wild-type (WT) αA- and αB-crystallins, each protein individually displayed a reduced level of activity, with the NH_2_-terminal regions showing maximum activity with respect to their WT crystallins.

## Methods

### Materials

Molecular weight protein markers and DNA markers were purchased from Invitrogen (Carlsbad, CA) and Promega (Madison, WI), respectively. Primers used in the study were obtained from Sigma-Aldrich (St. Louis, MO). Anti-Histidine-tagged mouse monoclonal primary antibody and goat anti-mouse IgG (H^+^L) horseradish peroxidase-conjugated secondary antibody were obtained from Calbiochem-EMD Biosciences (La Jolla, CA) and Thermo Scientific (Rockford, IL), respectively. Molecular biology-grade chemicals were purchased from either Sigma or Fisher Scientific (Fair Lawn, NJ), unless otherwise stated.

### Bacterial strains and plasmids

The *Escherichia coli* One Shot® TOP 10 cells and BL21 Star (DE3) bacterial strain were obtained from Invitrogen. TOP 10 cells were used for propagation and BL21 cells were used for expression. Plasmids containing WT αA- and αB-crystallin genes were already present in the laboratory.

### Generation of αA- and αB-crystallin constructs

A plasmid containing the human WT αA-crystallin gene in pET 100D TOPO vector (Invitrogen) [20] was used as a template for generating the desired constructs of αA-crystallin. The WT αB-crystallin gene [21] was subcloned into pET 100D TOPO vector (Invitrogen) and was used as a template for generating the desired constructs of αB-crystallin. Cloning in the pET 100 Directional TOPO vector added a six His-tag at the NH_2_-terminus of the protein, which allowed us to purify cloned proteins in a single affinity chromatographic step using a Ni^2+^- affinity column. PCR-based deletion was used to generate the desired constructs, using specific complimentary primer pairs (Table 1). The following constructs were generated by PCR-directed mutagenesis: (i) NH_2_-terminal domain consisting of residues no. 1–63 of αA and residues no. 1–66 of αB, (ii) Core domain consisting of residues no. 64–142 of αA and residues no. 67–146 of αB, and (iii) COOH-terminal extension consisting of residues no. 143–173 of αA and residues no. 147–175 of αB. Briefly, 25 ng of template was used under the following PCR conditions: pre-denaturation at 95 °C for 5 min, followed by 30 cycles of denaturation at 95 °C for 1 min, annealing at 62–68 °C for 45 s (depending on the T_m_ of the primers), and extension/elongation at 72 °C for 1 min, with a final extension at 72 °C for 10 min. PCR products were ligated into the pET 100 Directional TOPO vector as per the manufacturer’s instructions. Deletions at desired sites were confirmed by DNA sequencing (Genomics Core Facility of the University of Alabama at Birmingham, Birmingham, AL) at the transcriptional level, and by western blot analysis using an anti-His tag monoclonal antibody at the translational level. Positive clones were transformed into *E. coli* BL21 Star (DE3) cells, and selected using ampicillin.

**Table 1:** Oligonucleotide primers used for generation of individual domain constructs of αA- and αB-crystallins using PCR-based deletion.

**Mutant constructs**	**Direction**	**Primers (5′-3′)**
αA NTD	Fwd	CACCATGGACGTGACCATCCAGCACCCC
	Rev	TTACTCAGAGATGCCGGAGTCCAGCACGGT
αA CD	Fwd	CACCGTTCGATCCGACCGGGACAAGTTCGTC
	Rev	TTAACAGAAGGTCAGCATGCCATCGGCAGA
αA CTE	Fwd	CACCGGCCCCAAGATCCAGACTGGCCTGGAT
	Rev	TTAGGACGAGGGAGCCGAGGTGGGGTTCTC
αB NTD	Fwd	CACCATGGACATCGCCATCCACCACCCC
	Rev	TTATGAGAGTCCAGTGTCAAACCAGCT
αB CD	Fwd	CACCGAGATGCGCCTGGAAAAGGACAGG
	Rev	TTAATTCACAGTGAGGACCCCATCAGA
αB CTE	Fwd	CACCGGACCAAGGAAAGAGGTCTCTGGC
	Rev	CTATTTCTTGGGGGCTGCGGTGAC

### Expression and extraction of soluble proteins and proteins in inclusion bodies

PCR amplicons were transformed into *E. coli* BL21 Star (DE3) cells, as previously described, using a standard *E. coli* transformation technique [20]. Isopropyl-β-D-thio-galactoside (IPTG), at a final concentration of 1 mM, was added to overexpress the proteins, and cell cultures were incubated for 4 h at 37 °C. Cells were harvested, resuspended in lysis buffer (25 mM Tris-HCl [pH 7.8], 50 mM NaCl, 0.9% glucose, 1 mM EDTA, containing lysozyme [0.25 mg/ml] and protease inhibitor cocktail [Sigma]) and sonicated while kept on ice. DNA was degraded by treatment with DNase I (10 μg/ml) for 30 min on ice. The soluble fraction was separated by centrifugation at 8,000× g for 10 min at 4 °C, and the insoluble fraction was resuspended in detergent buffer (DB; 0.5 M NaCl, 1% [w/v] sodium deoxycholate, 1% NP-40, and 20 mM Tris-HCl, pH 7.5). Further, the detergent-soluble fraction was separated by centrifugation at 5,000× g for 10 min at 4 °C. The resultant pellet was washed with 0.5% Triton X-100 and centrifuged, as stated above, and the washing step was repeated as necessary to remove bacterial debris from the inclusion bodies. The final pellet was resuspended in denaturating binding buffer (DBB; [8M urea, 0.5 M NaCl, and 20 mM sodium phosphate, pH 7.8]).

### Purification of WT αA- and WT-αB-crystallins and their constructs

Depending on the presence of expressed proteins in soluble fractions or inclusion bodies (insoluble fractions), each protein was purified under native or denaturing conditions. All purification steps were performed at 4 °C, unless otherwise stated, including refolding steps. Proteins samples were adsorbed on an Invitrogen ProBond Ni^2+^-chelating column according to the manufacturer’s instructions. Under native conditions, the column was equilibrated and loaded with the protein sample using a native binding (NB) buffer (20 mM sodium phosphate containing 0.5 M NaCl, pH 7.8), washed with NB buffer containing 20 mM imidazole (pH 7.8), and eluted with NB buffer containing 250 mM imidazole (pH 7.8). Under denaturing conditions, the column was equilibrated with DBB, and following the application of desired protein preparation, the unbound proteins were eluted by a first wash with DBB; followed by a second and third wash with DBB at pH 6.0 and pH 5.3, respectively. Finally, the bound proteins were eluted with DBB containing 250 mM imidazole (pH 7.8).

Fractions recovered from Ni^2+^-affinity column chromatography under native or denaturing conditions were analyzed by SDS–PAGE [32]. Those purified under native conditions were dialyzed against 50 mM phosphate buffer (pH 7.8) at 4 °C, and stored at −20 °C until needed. Proteins purified under denaturing conditions were refolded using a previously published method [33] as briefly described below. Purity of WT αA- and αB-crystallins and their constructs was examined by SDS–PAGE and their identities were confirmed by western blot analyses [34] using an anti-His-tagged monoclonal antibody. Protein concentrations were determined by absorbance at 280 nm using a NanoDrop 2000 spectrophotometer (Thermo Scientific).

### Refolding of proteins purified under denaturing conditions

Proteins purified under denaturing conditions were refolded by 24 h dialysis at 4 °C against 50 mM sodium phosphate (pH 7.5) containing 1 mM DTT and decreasing urea concentrations from 8 M to 4 M, and finally in a urea-free phosphate buffer [33].

### Characterization of structural/functional properties of WT αA- and WT αB-crystallins and their constructs

The studies described below were performed with freshly purified preparations of αA- and αB-crystallins. Freezing and thawing of these preparations, which sometimes caused their precipitation, was avoided.

### Circular dichroism (CD) spectroscopy

The far-UV CD spectra of purified WT αA- and WT αB-crystallins and their constructs were recorded at room temperature over a range of 190 – 260 nm on a Jasco J815 CD spectrometer (Jasco, Easton, MD) using 0.2 mg/ml of protein in 50 mM sodium phosphate buffer (pH 7.8), as described previously [22]. A quartz cell of 0.5 mm path length was used, and the reported spectra are an average of five scans baseline-corrected for the buffer blank and smoothed. The secondary structural contents of WT proteins and constructs were determined by analysis of the CD spectra using the SELCON3 analysis program.

### ANS binding and fluorescence spectroscopy

Binding of a hydrophobic probe, 8-anilino-1-naphthalene sulfonate (ANS), to WT αA- and WT αB-crystallins and their constructs was measured by recording fluorescence emission spectra at 400 – 600 nm with excitation at 390 nm, as previously described [20,21]. For this, 15 μl of 0.8 mM ANS (dissolved in methanol) was added to 0.2 mg/ml of a protein in 50 mM sodium phosphate buffer (pH 7.8), mixed thoroughly, and incubated for 15 min at 37 °C before spectroscopy.

### Oligomer size determination by dynamic light scattering

A multiangle laser light scattering instrument (Wyatt Technology, Santa Barbara, CA) coupled to an HPLC system was used to determine the absolute molar mass of the WT proteins and their constructs. Prior to their analysis, protein samples in 50 mM sodium phosphate (pH 7.8) were filtered through a 0.22 μm filter. Results were acquired using 18 different angles, which were normalized with the 90° detector.

### Chaperone activity assay

To assess the ability of different α-crystallin constructs to prevent DTT-induced insulin aggregation, chaperone activity was determined by following a previously described method [21]. Aggregation of insulin by reduction with 20 mM DTT at room temperature, either in absence or at a 1:1 ratio of different αA- and αB-crystallin species in 1 ml reaction volumes containing 50 mM sodium phosphate (pH 7.8) was determined. Aggregation was monitored by measuring light scattering at 360 nm as a function of time using a Shimadzu UV-VIS scanning spectrophotometer (model UV2101 PC; Shimadzu, Columbia, MD) equipped with a six-cell positioner and a temperature controller (Shimadzu model CPS-260).

## Results

### Confirmation of site-specific deletions in αA- and αB-crystallin constructs

**Figure 1:**
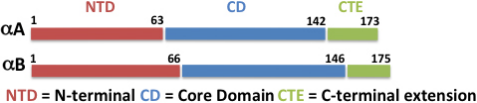
Schematic diagram showing the regions and residue numbers forming the NH_2_-terminal domain (NTD), core domain (CD), and COOH-terminal extension (CTE) of αA- and αB-crystallins in the current study. The residues spanned by each domain were as follows: αA NTD (residues no.1–63), αA CD (residues no. 64–142), αA CTE (residues no. 143–173), αB NTD (residues no. 1–66), αB CD (residues no. 67–146), and αB CTE (residues no. 147–175).

Recombinant WT αA and WT αB plasmids, present in our laboratory [20,21], were used as templates to generate constructs of individual α-crystallin domains (see Methods). The individual NH_2_-terminal domain, core domain, and COOH-terminal extension constructs of both αA- and αB-crystallins were generated using PCR-based deletion and are referred to as αA NTD, αA CD, αA CTE, αB NTD, αB CD, and αB CTE in the text (Figure 1). DNA sequencing confirmed the desired constructs: αA NTD (residues no. 1–63), αA CD (residues no. 64–142), αB NTD (residues no. 1–66), αB CD (residues no. 67–146), and αB CTE (residues no. 147–175).

### Expression and purification of WT α-crystallins and constructs

Expression of WT αA, WT αB, and their constructs was induced in the BL21 Star (DE3) expression cell line using 1 mM IPTG for 4 h, as previously described [20], and proteins were recovered in either the soluble fraction, insoluble fraction (inclusion bodies), or both fractions (Table 2). WT αA, WT αB, and αB CTE proteins were recovered in the soluble fraction, whereas αA NTD and αB NTD proteins were recovered in the insoluble fraction. These results were expected considering the hydrophobic nature of the NH_2_-terminal domain and hydrophilic nature of the COOH-terminal extension. However, the αA CD and αB CD proteins were recovered in both the soluble and insoluble fractions, suggesting their partial solubility property. The expression and solubility of individual proteins were confirmed by western blot analysis using a specific monoclonal antibody against a 6× His-tag epitope (data not shown).

**Table 2:** Presence of WT αA, WT αB, and the NH_2_-terminal, core domain, and COOH-terminal extension constructs in the soluble fraction and/or inclusion bodies.

**Crystallin species**	**Soluble fraction**	**Inclusion bodies**
WT αA	+	-
αA NTD	-	+
αA CD	+	+
αA CTE	ND*	ND*
WT αB	+	-
αB NTD	-	+
αB CD	+	+
αB CTE	+	-

+ Indicates presence of protein in given fraction on SDS–PAGE. *ND: not determined.

**Figure 2:**
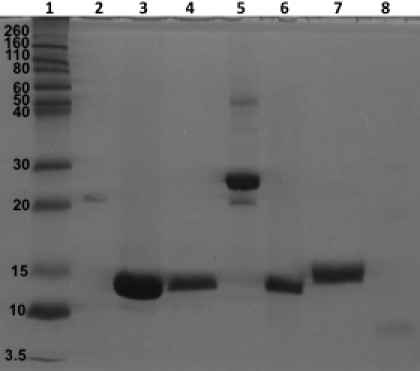
SDS–PAGE analysis of purified WT αA, WT αB and their individual domain constructs following purification. Protein purification was performed using Ni^2+^-affinity column chromatography (see Methods). Lane 1 – molecular weight marker; Lane 2 – WT αA; Lane 3 – αA NTD; Lane 4 – αA CD; Lane 5 – WT αB; Lane 6 – αB NTD; Lane 7 – αB CD; and Lane 8 – αB CTE. The protein band in each preparation showed immunoreactivity to anti-His tag monoclonal antibody (Results not shown).

Each protein was overexpressed in *E. coli* at 37 °C and purified to almost homogeneity, under native or denaturing conditions, using Ni^2+^-affinity columns (see Methods). On SDS–PAGE analysis, the molecular weights (M_r_) of purified His-tagged WT αA, WT αB, and their constructs ranged between 7 and 27 kDa (Figure 2). WT αA and WT αB showed a M_r_ of ~25–27 kDa (Figure 2, lanes 2 and 5), while the M_r_ of the NH_2_-terminal domain constructs (αA residues 1–63 and αB residues 1–66) were ~11–13 kDa, core domain constructs (αA residues 64–142 and αB residues 67–146) were ~13–14 kDa, and the COOH-terminal extension construct (αB residues 147–175) was ~7 kDa (Figure 2, lanes 3, 4, 6–8). The SDS–PAGE gel showed the highly purified nature of these proteins, and western blot analysis using a monoclonal anti-His antibody confirmed their identity as His-tagged α-crystallin proteins (data not shown). A 50 kDa band seen in lane 5 of Figure 2 was a dimer of WT αB as it and the monomer of the crystallin showed immunoreactivity with anti-His tagged monoclonal antibody.

### Comparative properties of individual α-crystallin domains and WT α-crystallins:

#### Circular dichroism spectral studies

**Figure 3:**
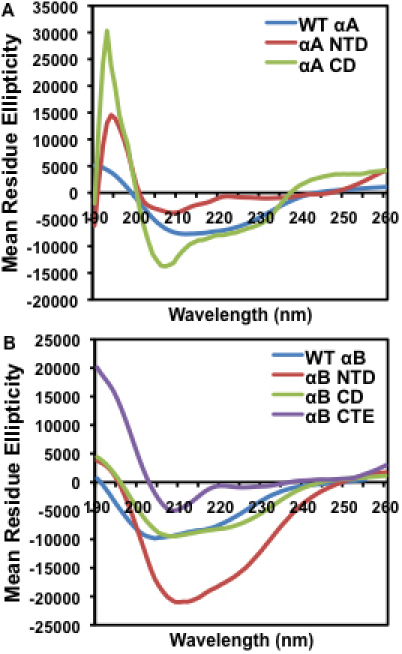
Far-UV CD spectra of WT αA, WT αB and their individual domain constructs. Spectra were recorded using protein preparations of 0.2 mg/ml, dissolved in 50 mM sodium phosphate buffer (pH 7.8), and a cell path length of 0.5 mm. The reported spectra are the average of 5 scans, corrected for the buffer blank, and smoothed. **A**: WT αA, NH_2_-terminal domain, and core domain constructs. **B**: WT αB, NH_2_-terminal domain, core domain, and COOH-terminal extension constructs.

To evaluate the effect of deletion/truncation of specific regions on the secondary structure of both αA and αB, far-UV CD spectra and secondary structural content of their individual regions were determined (Figure 3 and Table 3). As evident, the constructs exhibited varied CD spectra (Figure 3A,B) compared to WT αA- and WT αB-crystallins. Based on secondary structural content, as determined using the SELCON3 analysis software, WT αA exhibited 21.6% α-helix, 46.6% β-sheet, 13.4% β-turn, and 17.6% random coil. In contrast, the αA NTD construct showed a marked difference in secondary structure with 80.5% α-helix, 7.9% β-sheet, 3.2% β-turn, and 8.4% random coil, suggesting that the NH_2_-terminal domain of αA-crystallin by itself assumes a more helical structure (Figure 3A and Table 3). The αA CD construct varied slightly from WT αA with 37.9% α-helix, 42.7% β-sheet, 10.7% β-turn, and 9.6% random coil, suggesting that αA CD alone becomes slightly more helical and retains a secondary structure relatively similar to WT (Figure 3A and Table 3). Based on SELCON3 analysis, WT αB exhibited 19.3% α-helix, 48.7% β-sheet, 12.4% β-turn, and 19.6% random coil. However, αB NTD differed in secondary structural content with 29.9% α-helix, 41.3% β-sheet, 9.1% β-turn, and 19.4% random coil. The increase in α-helical content and slight decrease in β-sheet of αB NTD produced a readily visible difference in spectra compared to WT αB (Figure 3B). Like αA CD, αB CD also exhibited only a slight difference in its CD spectra relative to WT αB (Figure 3B), as the latter showed 20.6% α-helix, 48.1% β-sheet, 14.3% β-turn, and 18.7% random coil (Table 3). Among the αB-crystallin constructs, αB CTE showed the most marked difference in secondary structure compared to WT, with 5.9% α-helix, 66.6% β-sheet, 4.3% β-turn, and 23.5% random coil. αB CTE also exhibited more random coil structure than all other constructs (Table 3). This noticeable decrease in α-helical content and increase in β-sheet suggest that, by itself, the COOH-terminal extension of αB-crystallin has more β-sheet structure. Taken together, the results suggested that, individually, the domains flanking the α-crystallin core domain display greater alterations in secondary structure than the core domains when compared to their respective WT proteins. The most notable changes were that the NH_2_-terminal domain construct of αA-crystallin showed greater α-helical content compared to WT αA, while the COOH-terminal extension of αB-crystallin showed a substantial decrease in α-helical content and increase in β-sheet content relative to WT αB.

**Table 3:** Secondary structural content of WT αA, WT αB and their individual domain constructs. Percentages were determined by analysis of the Far-UV spectra (Figure 3) using the SELCON3 analysis program.

**Crystallin species**	**α-Helix (±1%)**	**β-Sheet (±1%)**	**β-Turn (±1%)**	**Random coil (±1%)**
WT αA	21.6	46.6	13.4	17.6
αA NTD	80.5	7.9	3.2	8.4
αA CD	37.9	42.7	10.7	9.6
WT αB	19.3	48.7	12.4	19.6
αB NTD	29.9	41.3	9.1	19.4
αB CD	20.6	48.1	14.3	18.7
αB CTE	5.9	66.6	4.3	23.5

#### Surface hydrophobicity

**Figure 4:**
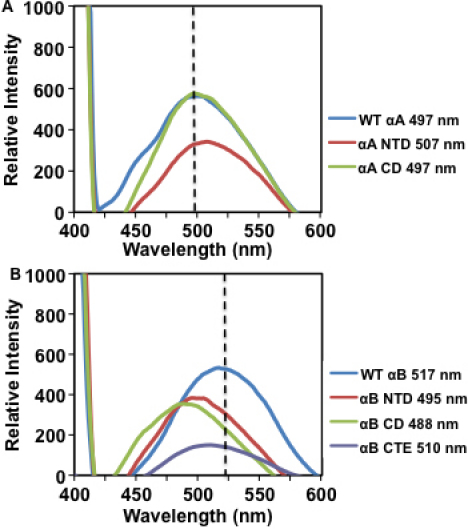
Fluorescence spectra of WT αA, WT αB and their individual domain constructs following ANS binding. Fluorescence spectra were recorded by excitation at 390 nm and emission from 400 to 600 nm, with protein preparations (0.2 mg/ml) mixed with 15 μl of 0.8 mM ANS (dissolved in methanol) and incubated at 37 °C for 15 min. **A**: WT αA, NH_2_-terminal domain, and core domain constructs. **B**: WT αB, NH_2_-terminal domain, core domain, and COOH-terminal extension constructs. Dotted lines indicate the maximum peak wavelength (λ_max_) of the respective WT α-crystallins, used to determine whether a blue or red shift in wavelength occurred.

Changes to the secondary structure of a protein likely affect its tertiary structural conformation as well. Previous studies have implicated the exposed hydrophobic surfaces of α-crystallins in the binding of target proteins during chaperone function [7,33,35]. In light of the altered secondary structures of the individual domains of αA- and αB-crystallins, we investigated surface hydrophobicity binding, among WT proteins and their constructs, by using a hydrophobic fluorescence probe, ANS (Figure 4). ANS is a useful probe for assaying surface hydrophobicity because it is non-fluorescent in aqueous solutions, but fluoresces when bound to hydrophobic surfaces, so its fluorescence correlates to its binding. On ANS binding, WT αA exhibited fluorescence with λ_max_ peak at 497 nm, and similarly, αA CD showed a fluorescence peak at 497 nm with identical intensity. However, with a peak at 507 nm, αA NTD exhibited a 10 nm red shift relative to WT αA, and it was coupled with a decrease in fluorescence intensity (Figure 4A). The results suggest that, relative to WT αA, the N-terminal domain construct showed a relatively relaxed structure with a greater exposure of hydrophobic surfaces but a decrease in binding intensity, while the αA core domain retained its surface hydrophobicity property. Upon binding to ANS, WT αB exhibited a fluorescence peak with λ_max_ at 517 nm. However, αB NTD, CD, and CTE all showed a blue shift compared to WT αB, as well as reduced fluorescence intensity, with peaks at 495 nm, 488 nm, and 510 nm, respectively (Figure 4B). The differences in λ_max_ peaks of the αB-crystallin constructs relative to WT αB were as follows: a 22 nm shift in the αB NTD peak, a 29 nm shift in αB CD peak, and a 7 nm shift in αB CTE peak. Of all the constructs, the COOH-terminal extension construct showed a substantial decrease in fluorescence intensity, compared to WT αB. The results suggest that, compared to WT, the individual αB-crystallin domain constructs displayed relatively compact structures with both decreased exposure of hydrophobic surfaces and decreased binding intensity. Taken together, shifts in fluorescence peaks and changes in intensity suggest changes in the microenvironments surrounding the hydrophobic residues and imply changes to the tertiary structures of these proteins compared to their WT crystallins.

### Determination of molecular mass by dynamic light scattering

To determine whether the individual crystallin domain constructs were able to oligomerize, the molecular masses of WT αA, WT αB and their constructs were determined by multi-angle light scattering (MALS) analysis (Wyatt Technology, Santa Barbara, CA). Table 4 shows the molecular mass of each protein. Compared to WT αA, which had a mass of 6.8×10^5^ D, the αA NTD and αA CD constructs showed a mass of 1.6×10^4^ D and 3.0×10^4^ D, respectively. Individually, the NH_2_-terminal domain and core domain of αA-crystallin form smaller oligomers, though the core domain forms slightly larger oligomers than the NH_2_-terminal domain. Similar to WT αA, WT αB had a mass of 5.8×10^5^ D. Compared to WT αB, αB NTD displayed a mass of 2.6×10^3^ D, however, both αB CD and αB CTE showed increases in mass of 8.4×10^7^ D and 1.0×10^6^ D, respectively. Unlike αA, the NH_2_-terminal domain of αB forms smaller oligomers, while the core domain and COOH-terminal extension both form much larger oligomers compared to WT αB.

**Table 4:** Molar mass determination of WT αA, WT αB and their individual domain constructs using the dynamic light scattering method (MALS).

**Crystallin species**	**Molecular mass (Da)**
WT αA	6.8×10^5^
αA NTD	1.6×10^4^
αA CD	3.0×10^4^
WT αB	5.8×10^5^
αB NTD	2.6×10^3^
αB CD	8.4×10^7^
αB CTE	1.0×10^6^

### Chaperone activity of WT α-crystallins and individual domain constructs

**Figure 5:**
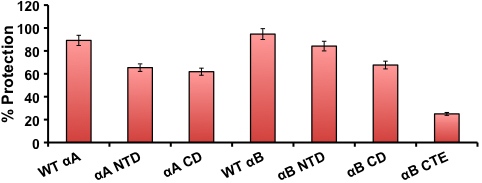
Chaperone activity comparison of WT αA and αB and their individual domain constructs. The chaperone activity, calculated as % protection, was assayed by measuring DTT-induced insulin (100 μg) aggregation in the presence of chaperone/insulin ration (1:1) at 25 °C. Error bars=Percent Error (+/− 1%).

To determine if the individual α-crystallin domains retain functionality, the chaperone activity of these constructs was assayed. Insulin along with other target proteins such as alcohol dehydrogenase, lysozyme, and lactalbumin has provided comparable results in previously published reports [13,25,29]. Thus, the chaperone activities of WT αA and WT αB and their individual domain constructs were determined using insulin (100 μg) as the target protein in a 1:1 ratio. Light scattering at 360 nm was used to measure aggregation of the insulin B chain by reduction with DTT in the presence and absence of chaperone proteins. Chaperone activity was represented as the percent protection provided by the crystallins (Figure 5). WT αA exhibited about 90% protection against DTT-induced insulin aggregation, whereas both αA NTD and αA CD constructs showed a decrease in chaperone activity, providing about 65% and 60% protection, respectively. Similar to WT αA, WT αB exhibited about 95% protection against insulin aggregation. However, both αB NTD and αB CD constructs showed decreased chaperone activity compared to WT. αB NTD showed the greatest protection at about 85%, while αB CD showed about 70%. αB CTE provided only minimal protection to insulin at about 25%. In general, among the constructs, chaperone activity decreased as follows: NTD>CD>CTE. More specifically, chaperone activity across all proteins decreased in the following order: WT αB> WT αA> αB NTD> αB CD> αA NTD> αA CD> αB CTE (Figure 5). Taken together, the results suggested that isolating the individual domains of both αA- and αB-crystallins, particularly the NH_2_-terminal domains, affected the chaperone activity of the proteins, but did not completely deplete their function.

## Discussion

The purpose of the present study was to understand the biophysical and functional aspects of the NH_2_-terminal domain, core domain, and COOH-terminal extension of both αA- and αB-crystallins, with the intent to find molecules (peptides) of αA and αB which can assist in the correct folding of other proteins or in sequestering misfolded/improperly folded proteins, and also eventually act as a targeting drug. The present study was the first step toward our long-term objective. To accomplish this, individual constructs of NH_2_-terminal domain, core domain, and COOH-terminal extension of both αA- and αB-crystallins were generated and used for comparative structural and functional analyses to ascertain which region is most pertinent to chaperone function. Two constructs of αA-crystallin (i.e., αA NH_2_-terminal domain [αA NTD, residues 1–63] and core domain [αA CD, residues 64–142]) and three constructs of αB-crystallin (i.e., αB NH_2_-terminal domain [αB NTD residues 1–66], core domain [αB CD, residues 67–146], and COOH-terminal extension [αB CTE, residues 147–175]) were generated. One construct, αA COOH-terminal extension [αA CTE, residues 143–173] could not be successfully generated even after several attempts and using variable conditions. This could be due to one of the templates being technically difficult to amplify, with high GC content or other structures that cause DNA polymerase to stop or pause. Our failure could also be due to other reasons, which are presently unknown to us.

The reason for selectively using the three recombinant regions (i.e., NH_2_-terminal domain, core domain, and COOH-terminal extension) of αA- and αB-crystallins for the comparative structural and functional studies was because several past reports have described effects of either partial deletion or mutation in the three regions, but never examined the properties of each individual region relative to their WT proteins. Further, based on preliminary experimental evidence in the literature, a persistent belief exists that the chaperone proteins/peptides could be the key to treating a range of diseases related to protein misfolding, such as cataract, Alzheimer and Parkinson. Our experimental approach was similar to several past reports that considered the three regions of the two crystallins as modules to determine their affect on crystallin structural stability and function. Some such examples are as listed: (1) Merck et al. [36] studied recombinant NH_2_-terminal domain and COOH-terminal domain of αA-crystallin, and concluded that the interactions leading to aggregation of αA-crystallin subunits are mainly located in the NH_2_-terminal half of the chain. (2) Merck et al. [37] also compared the COOH-terminal domain and tail region of αA crystallin from rat, bovine αB crystallin and mouse HSP25, to show that the COOH-terminal domain of αA formed dimers and tetramers, but corresponding regions of αB and HSP25 formed larger aggregates and the COOH-terminal domain lacked heat protection activity. (3) The swapping of the COOH-terminal extension of αA-crystallin to the COOH-terminal extension of αB led to enhanced chaperone activity of the latter [29], suggesting that in addition to the solubilizer property of this region, it also plays a crucial role in structural stability and chaperone activity. (4) Kokke et al. [38] used Hsp12.2 from roundworm *Caenorhabditis elegans* to study the role of the α-crystallin domain. Although Hsp12.2 forms oligomers like other sHSPs, it lacks chaperone activity. In spite of addition of the NH_2_-terminal domain and COOH-terminal extension of human αB-crystallin to the α-crystallin domain of Hsp12.2, the chaperone activity was not restored. The results suggested that proper synergism between different regions is necessary for chaperone activity. (5) To determine the role of NH_2_- and COOH-terminal sequences in assembly and function of sHSPs, Ghosh et al. [39] used two deletion mutants, ∆41–58 (lacking residues 41–58 of the NH_2_-terminal domain) and ∆155–165 (lacking residue no. 155–165). These two regions were identified as interacting regions in formation of αB-crystallin oligomers [40]. Oligomers of the two deletion mutants were larger and more polydisperse relative to WT αB. The ∆41–58 mutant showed the same level of chaperone activity as WT αB whereas the ∆155–165 mutant lost its chaperone activity and solubility. (6) Smulders et al. [41] reported that on insertion of either charged residues (Lys, Arg and Asp) or a hydrophobic residue (Trp) in the COOH-terminal extension of αA, only the insertion of the hydrophobic residue seriously disturbed the structure and function of the crystallin. (7) Peptides as minichaperones of both αA- (residue no. 78–88 [in the α-crystallin domain region]) and αB- (residue no. 73–92 [in the α-crystallin domain region]) crystallins have been extensively studied for their structural and functional properties [12,13]. Because these are functional elements (peptides of both αA and αB-crystallins), they suggest a lesser requirement of both NH_2_-terminal domain and COOH-terminal extension of the crystallins in their chaperone activity.

Each of the constructs in our study was confirmed by DNA sequencing. Similarly, the specific protein products of the constructs were confirmed by their expected M_r_’s following SDS–PAGE analysis, and by immunoreactivity to an anti-His-tag antibody during western blot analysis. Each of the His-tagged proteins was purified by one step Ni^2+^-affinity column chromatography, and SDS–PAGE analysis showed their recovery in highly purified forms (Figure 2).

The following were the major findings of the study: (1) In contrast to the high content of 46.6% β-sheet and only 21.6% α-helix in WT αA, αA NTD showed high α-helix (80.5%) and substantially low (7.9%) β-sheet content. Similarly, the αA CD construct showed 37.9% α-helix and 42.7% β-sheet content, suggesting that both the NH_2_-terminal domain and core domain alone of αA-crystallin assume a more helical structure than WT αA (Figure 3A and Table 3). (2) Relative to 19.3% α-helix and 48.7% β-sheet content of WT αB, αB NTD also showed a higher α-helical content of 29.9%, and a lower β-sheet content of 41.3%. However, αB CD showed 20.6% α-helix and 48.1% β-sheet, which was similar to WT αB-crystallin. In contrast, αB CTE showed only 5.9% α-helix and 66.6% β-sheet, which was the most marked difference in secondary structure compared to WT αB. (3) On determination of hydrophobicity by ANS-binding, WT αA and αA CD exhibited λ_max_ fluorescence peaks at 497 nm with identical intensity, whereas αA NTD exhibited a 10 nm red shift with a λ_max_ peak at 507 nm, suggesting that the secondary structure was relatively relaxed with greater exposed hydrophobic surfaces. On a similar ANS binding, WT αB showed λ_max_ at 517 nm, whereas αB NTD, αB CD, and αB CTE showed blue shifts compared to WT with λ_max_ peaks at 495 nm, 488 nm, and 510 nm, respectively. Therefore, relative to WT αB, the differences in λ_max_ peaks of the αB-crystallin constructs were as follows: a 22 nm shift in the αB NTD peak, a 29 nm shift in the αB CD peak, and a 7 nm shift in the αB CTE peak. Together, the results suggest that compared to WT, the individual αB constructs displayed relatively compact structures with a decrease in both exposure of hydrophobic surfaces and binding intensity. (4) The MALS results showed that relative to a M_r_ of 6.8×10^5^ Da of WT αA oligomers, its constructs also oligomerized but exhibited lower molecular mass (i.e., αA NTD and αA CD M_r_ of 1.6×10^4^ Da and 3.0×10^4^ Da, respectively). Similarly, relative to the M_r_ of 5.8×10^5^ Da of WT αB, the αB NTD construct showed a substantially lower M_r_ of 2.6×10^3^ Da, while the other two constructs showed higher M_r_ (i.e., αB CD and αB CTE displayed 8.4×10^7^ Da and 1.0×10^6^ Da, respectively). Therefore, relative to the NH_2_-terminal domain of αB, both the core domain and COOH-terminal extension of αB formed much larger oligomers, which were even bigger than the oligomers of WT αB. (5) WT αA and WT αB exhibited almost the same levels of about 90% protection against DTT-induced insulin aggregation. However, both NH_2_-terminal and core domain constructs of αA also showed 65% and 60% protection, respectively. Similarly, αB NTD, αB CD and αB CTE exhibited protection at about 85%, 70%, and 25%, respectively. Together, the results show a slightly greater chaperone activity of the NH_2_-terminal domain of αB relative to NH_2_-terminal domain of αA, but similar levels of activity of core domain of the two crystallins. The COOH-terminal extension construct of αB exhibited substantially low (25%) chaperone activity relative to all other regions of both αA and αB-crystallins. We anticipate a similar low level of chaperone activity of αA CTE as has been reported previously [37].

The above differences in the structural and functional properties in the three regions of αA and αB were seen in spite of their origin via gene duplication and having ~57% sequence homology [42]. Some other examples showing differences in the two crystallins include: recombinant αA- and αB-crystallins differ in their secondary and tertiary structures, and relative to αA, αB showed a greater hydrophobicity and fourfold more chaperone activity [43]. Other reports showed that αA-crystallin was more stable to gamma irradiation relative to αB [44], and although αA- and αB-crystallins can each form oligomers independently, together or with other crystallins, their interactions with each other were threefold greater than their interactions with βB2- and γC-crystallins [45]. Both crystallins also exhibit varied expression in different diseases. While αA is lens specific, αB-crystallin is widely expressed in other tissues, most prominently in astrocytes [46] and muscles [47]. αB has also been detected in the brain and associated with neurologic diseases such as Alzheimer [48], Parkinson [49], Creutzfeldt-Jakob disease [50], Alexander disease [51] and diffuse Lewy body disease [52].

As stated above, like other sHSPs, α-crystallin also contains a highly conserved sequence of 80–100 residues called the α-crystallin domain [2,29]. Based on similarities with the structures of other HSPs, it is believed that the NH_2_-terminal regions of both αA- and αB-crystallins form independently folded domains, whereas the COOH-terminal region is flexible and unstructured [2,29]. Our CD spectra results also suggest that the NH_2_-terminal domain of αA indeed formed an independently folded domain with high content of α-helix (80.5%) and low β-sheet (7.9%) compared to 46.6% β-sheet and 21.6% α-helix in WT αA. Similarly, αB NTD with 29.9% α-helix and 41.3% β-sheet also exhibited distinct secondary structure compared to WT αB with 19.3% α-helix, and 48.7% β-sheet. Because of the absence of data regarding the secondary structure of αA CTE, we could only compare the secondary structural data of αB CTE (5.9% α-helix, 66.6% β-sheet) with WT αB (19.3% α-helix, 48.7% β-sheet). The results show that αB CTE assumes a greater β-sheet structure relative to WT αB. αA NTD showed drastic differences in secondary structure because of significant increase in α-helical content and concurrent decrease in β-sheet content. The results of altered secondary structures of NH_2_-terminal domains of αA and αB compared to their WT proteins were also supported by their ANS binding results and molecular mass determined by the MALS method. Relative to WT αA (λ_max_ at 497 nm), αA NTD exhibited a λ_max_ peak at 507 nm with a 10 nm red shift whereas, compared to WT αB (λ_max_ at 517 nm), αB NTD showed λ_max_ of 495 nm with a 22 nm blue shift. Therefore, both αA NTD and αB NTD exhibited altered hydrophobicity relative to their WT proteins, but the former acquired a relatively relaxed structure and the latter a compact structure. Additionally, relative to molecular mass of 6.8×10^5^ Da of WT αA and 5.8×10^5^ Da of WT αB, both αA NTD and αB NTD exhibited oligomers with mass of 1.6×10^4^ Da and 2.6×10^3^ Da, respectively. The lower molecular mass of αB NTD compared to αA NTD supports their blue and red spectral shifts during ANS binding, respectively. On ANS binding, both WT αA and αA CD exhibited λ_max_ fluorescence peaks at 497 nm with identical intensity, however, the molecular mass of αA CD (3.0×10^4^ D) was significantly lower than that of WT αA (6.8×10^5^ D). Both αB CD and αB CTE showed a blue shift with λ_max_ of 488 and 510 nm, respectively. These results clearly showed changes in the microenvironment of hydrophobic patches of αB CD and αB CTE. However, in spite of compact structure, both αB CD and αB CTE displayed higher molecular mass of 8.4×10^7^ D and 1.0×10^6^ D, respectively, compared to WT αB. Together, the results show a greater tendency of αB CD and αB CTE to form oligomers of bigger sizes compared to those αA NTD, αB NTD and αACD. Further, interestingly, each region of both αA and αB could form oligomers in the same manner as full-length α-crystallins.

The present study showed that all three regions of both αA- and αB-crystallins are involved in varied levels of chaperone activity. While both WT αA and WT αB exhibited almost the same levels (~90%) protection against DTT-induced insulin aggregation, the higher level of chaperone activity of the NH_2_-terminal domains of both crystallins relative to their core domains and COOH-terminal extension suggest that it is most relevant among the three regions for the chaperone activity. This is supported by previous reports showing that, primarily, the residues within the NH_2_-terminal domain or near it are involved in chaperone substrate binding, i.e., residues 70–88 of αA-crystallin [12], as well as residues 73–92 of αB-crystallin [13]. Similarly, residues 54–61 of αB-crystallin are essential for its chaperone activity although unessential for target protein binding [14].

Reports have suggested that, as in other sHSPs, the NH_2_-terminal domain of αA-crystallin is important for chaperone activity, self-assembly into oligomers, and structural stability. Our previous report has shown that the deletion of the NH_2_-terminal domain of αA resulted in altered structure with properties such as increased hydrophobic patches, β-sheet content, and subunit exchange rate with WT-αB, but reduced oligomer mass and chaperone activity [22]. Residues 12–21 and 70–88 in the NH_2_-terminal domain of αA were identified as substrate binding sites [12]. Similarly, two bis-ANS binding sites at residues 50–54 and 79–99 were also identified [53]. Deletion of 1–63 amino acid residues in bovine α-crystallin resulted in the formation of only a tetrameric species [54], suggesting severely diminished oligomerization property. This is consistent with the peptide scan results, which showed that residues 42–57 and 60–71 of αA play a role in oligomerization and subunit interactions [55].

Our study showed little chaperone activity in the COOH-terminal extension of αB-crystallin, which is in parallel with previous studies which showed that on the removal of NH_2_-terminal residues (partial or 1–56 residues of the NH_2_-terminus) and COOH-terminal extension residues (partial or 32–34 residues of the COOH-terminus) of αA- and αB-crystallins, the proteins showed improper folding [2], reduced chaperone activity [22], and formation of trimers or tetramers [53-55].

In summary, we have taken an alternative approach in this study than the previously published reports that examined whether mutation or deletion of amino acids in any of the three regions of both αA- and αB-crystallins have effects on their structural and functional properties. The most intriguing finding of the present study was that although the three different regions (i.e., NH_2_-terminal domain, core domain, and COOH-terminal extension) of both αA- and αB-crystallins have different secondary structures, surface hydrophobicity and oligomerization properties, they individually retain variable levels of chaperone activity.
